# ﻿Two new species of the genus *Cheiracanthium* C. L. Koch, 1839 (Araneae, Cheiracanthiidae) from China

**DOI:** 10.3897/zookeys.1200.123214

**Published:** 2024-05-07

**Authors:** Zhaoyi Li, Feng Zhang

**Affiliations:** 1 Key Laboratory of Zoological Systematics and Application of Hebei Province, College of Life Sciences, Hebei University, Baoding, Hebei 071002, China Hebei University Baoding China; 2 Hebei Basic Science Center for Biotic Interaction, Hebei University, Baoding, Hebei 071002, China Hebei University Baoding China

**Keywords:** COI, description, DNA barcode, long-legged sac spider, taxonomy

## Abstract

Two species of the long-legged sac spider genus *Cheiracanthium* C. L. Koch, 1839 collected from China are diagnosed and described as new to science: *Cheiracanthiumbannaensis***sp. nov.** (♂♀) from Yunnan Province and *C.bifurcatum***sp. nov.** (♂♀) from Xinjiang Uyger Autonomous Region. Photos of the habitus and copulatory organs are given. In addition, DNA barcode information of the two new species is provided.

## ﻿Introduction

The genus *Cheiracanthium* C. L. Koch, 1839 is widely known and mainly distributed in the Old World (World Spider Catalogue; WSC 2024). Compared to other genera in Cheiracanthiidae Wagner, 1887, *Cheiracanthium* is the largest, accounting for 60% of the species diversity (220 out of 369 species described in the family) (WSC 2024). Members of *Cheiracanthium* are known as long-legged sac spiders, as they have long and slender legs, and build sac-like silk nests on plant leaves (Lotz 2007a).

Although several studies on *Cheiracanthium* have been published in the last few years (Deeleman-Reinhold 2001; Lotz 2007a, 2007b, 2011, 2014, 2015; Chen and Huang 2012; Bayer 2014; Zhang et al. 2018, 2020; Li and Zhang 2019, 2020, 2023, 2024; Esyunin and Zamani 2020; Dippenaar-Schoeman et al. 2021), the global diversity of this genus is still insufficiently known, and there are likely many other, as yet undiscovered species. Currently, 47 species of *Cheiracanthium* have been recorded from China, of which 12 species are known based on a single female (8) or male (4) (WSC 2024). Therefore, the identification of species and correct sex matching in *Cheiracanthium* are often challenging.

In the present paper, two new species of *Cheiracanthium* from China are recognized and described here: *Cheiracanthiumbannaensis* sp. nov. and *C.bifurcatum* sp. nov. In addition, the DNA barcode gene, cytochrome c oxidase subunit I (COI) of new species is given, as DNA information is useful for the identification of species and for correctly matching sexes (Lo et al. 2021; Li and Zhang 2023).

## ﻿Material and methods

All specimens were preserved in 75% ethanol and examined and measured under a Leica M205A stereomicroscope. Photographs were taken using an Olympus BX51 microscope equipped with a Kuy Nice CCD camera and were imported into Helicon Focus v.7 for stacking. Final figures were retouched using Adobe Photoshop 2020. All measurements are given in millimeters. Leg measurements are shown as: total length (femur, patella, tibia, metatarsus, tarsus). Epigynes were removed and cleared in a pancreatin solution. All specimens studied are deposited in the Museum of Hebei University (**MHBU**), Baoding, China.

Morphological terminology follows Zhang et al. (2020) and Li and Zhang (2023). The following abbreviations are used: A, atrium; AER, anterior eye row; ALE, anterior lateral eyes; AME, anterior median eyes; AME–ALE, distance between AME and ALE; AME– AME, distance between AMEs; C, conductor; CD, copulatory duct; CF, cymbial fold; CO, copulatory opening; CS, cymbial spur; DTA, dorsal tibial apophysis; E, embolus; FD, fertilisation duct; MA, median apophysis; MOA, median ocular area; PER, posterior eye row; PLE, posterior lateral eyes; PME, posterior median eyes; PME–PLE, distance between PME and PLE; PME–PME, distance between PMEs; RTA, retrolateral tibial apophysis; S, spermatheca.

A DNA barcode was also obtained for species delimitation and matching of different sexes. A partial fragment of the mitochondrial cytochrome oxidase subunit I (CO1) gene was amplified and sequenced using the primers LCO1490/ HCO2198 (Folmer et al. 1994). For additional information on extraction and amplification see Li and Zhang (2023). All PCR products were purified and sequenced at Sangon Biotech (Shanghai, China) Co., Ltd.

Sequence alignments were carried out using Mafft v.7.313 (Katoh and Standley 2013) with the L-INS-I strategy and checked for the presence of stop codons of COI by translating them into amino acid sequence using Geneious Prime (Kearse et al. 2012). Ambiguously aligned positions were culled using trimAl v.1.2 (Capella-Gutierrez et al. 2009) with default parameters. The pairwise genetic distances (Kimura two-parameter [K2P]) were calculated using MEGA v.11 (Tamura et al. 2021) to assess the genetic differences.

## ﻿Results

### ﻿DNA barcodes

All sequences were deposited in GenBank. The accession numbers of the generated DNA barcodes are provided in Table 1. The K2P genetic distance of intraspecific and interspecific nucleotide divergences of *C.bannaensis* sp. nov. and *C.bifurcatum* sp. nov. are shown in Table 2.

**Table 1.** Voucher specimen information.

Species	Voucher code	Sex	GenBank accession number	Collection localities
*C.bannaensis* sp. nov.	ZYL599	♂	PP493004	China, Yunnan
	ZYL600	♀	PP493005	China, Yunnan
	ZYL601	♀	PP493006	China, Yunnan
	ZYL602	♀	PP493007	China, Yunnan
	ZYL603	♀	PP493008	China, Yunnan
*C.bifurcatum* sp. nov.	ZYL604	♂	PP493009	China, Xinjiang
	ZYL605	♀	PP493010	China, Xinjiang

**Table 2.** Intraspecific and interspecific nucleotide divergences for *C.bannaensis* sp. nov. and *C.bifurcatum* sp. nov. using the Kimura two-parameter model.

Species	ZYL599	ZYL600	ZYL601	ZYL602	ZYL603	ZYL604	ZYL605
*C. bannaensis_*ZYL599							
*C. bannaensis_*ZYL600	0.0061						
*C. bannaensis_*ZYL601	0.0030	0.0061					
*C. bannaensis_*ZYL602	0.0183	0.0183	0.0185				
*C. bannaensis_*ZYL603	0.0183	0.0152	0.0153	0.0184			
*C. bifurcatum_*ZYL604	0.1440	0.1401	0.1452	0.1478	0.1382		
*C. bifurcatum_*ZYL605	0.1440	0.1401	0.1452	0.1478	0.1382	0.0000	

The intraspecific genetic distance ranged from 0 to 1.85%, and the interspecific genetic distance ranged from 13.82% to 14.78%. The maximum intraspecific distances were much lower than the minimum interspecific distances. The results of Kimura two-parameter genetic distances confirm the correct matching of male and female of two new species.

### ﻿Taxonomy

**﻿Family Cheiracanthiidae Wagner, 1887**

#### ﻿Genus Cheiracanthium

Taxon classificationAnimaliaAraneaeCheiracanthiidae

C. L. Koch, 1839

1A4540D6-C752-5962-B904-D3BAFAEE8E25

##### Type species.

*Araneapunctoria* Villers, 1789, by subsequent designation.

#### ﻿ Cheiracanthium bannaensis sp. nov.

Taxon classificationAnimaliaAraneaeCheiracanthiidae

4E90FA63-8091-52CB-9D6A-EC2341420344

https://zoobank.org/BB44B67A-A827-45C5-B55B-9BC143B9165A

**Figure 1.**
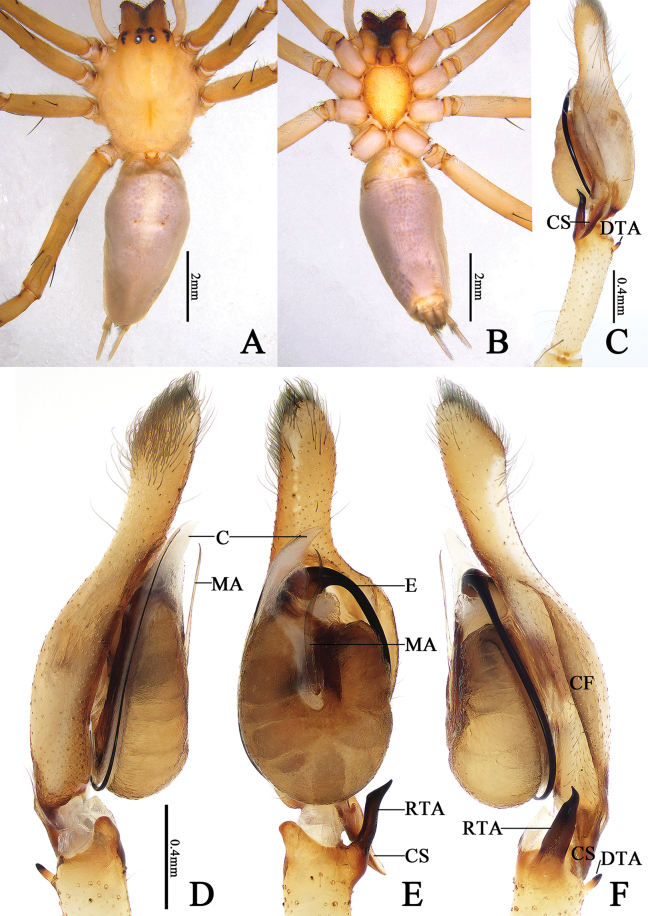
*Cheiracanthiumbannaensis* sp. nov., male holotype (ZYL599). **A** habitus, dorsal view **B** same, ventral view **C, F** left palp, retrolateral view **D** same, prolateral view **E** same, ventral view. Abbreviations: C = conductor, CF = cymbial fold, CS = cymbial spur, DTA = dorsal tibial apophysis, E = embolus, MA = median apophysis, RTA = retrolateral tibial apophysis.

**Figure 2.**
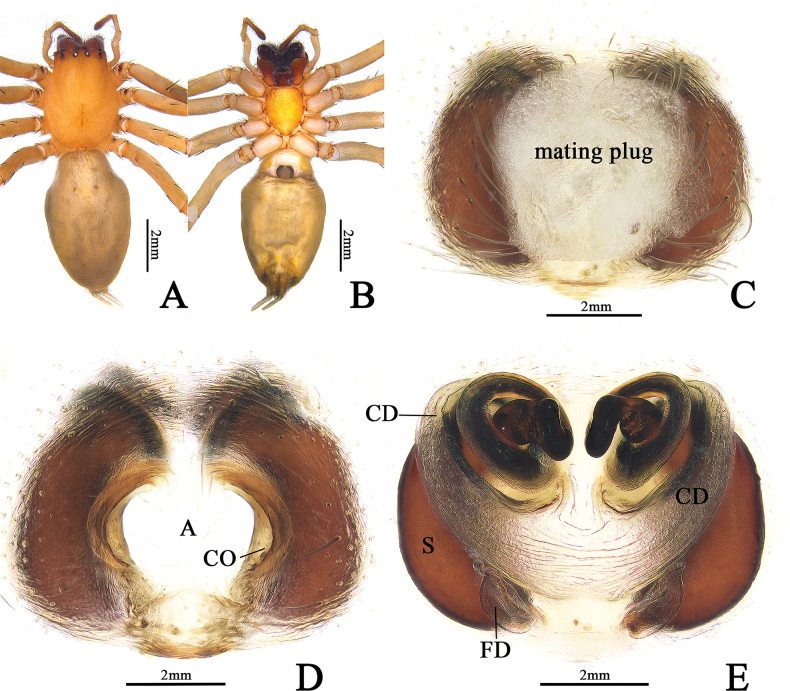
*Cheiracanthiumbannaensis* sp. nov., female holotype (ZYL600). **A** habitus, dorsal view **B** same, ventral view **C** epigyne, intact, ventral view **D** epigyne, cleared, ventral view **E** vulva, dorsal view. Abbreviations: A = atrium, CD = copulatory duct, CO = copulatory opening, FD = ertilisation duct, S = spermatheca.

**Figure 3.**
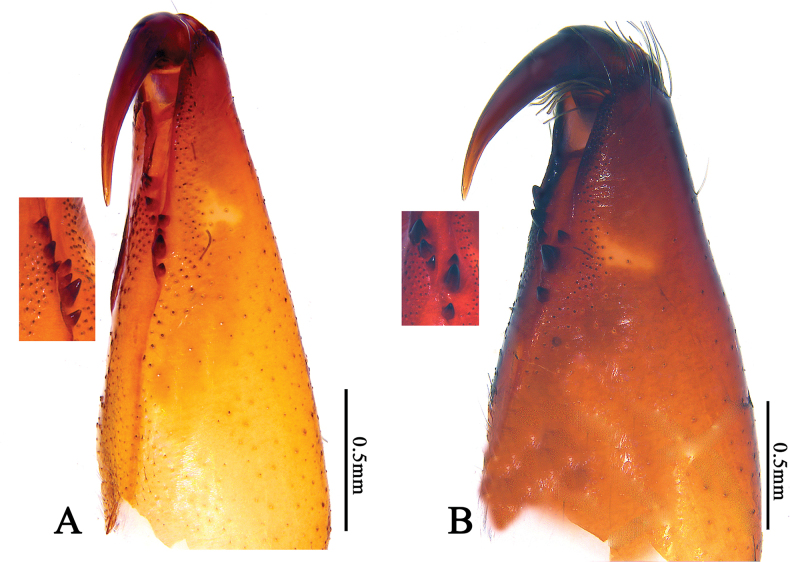
Right chelicerae of *Cheiracanthiumbannaensis* sp. nov. **A** male, retrolateral view **B** female, retrolateral view.

Figs 1 , 2 , 3 Chinese name: 版纳红螯蛛

##### Type material.

***Holotype*** ♂ (ZYL599), China: Yunnan Province, Xishuangbanna Dai Autonomous Prefecture, Menghai County, Alu Xinzhai, 21.869847°N, 100.460790°E, 1581 m elev., 11.VI.2022, leg. Zhaoyi Li. ***Paratype***: 4♀ (ZYL600–ZYL603), same data as holotype.

##### Etymology.

The species name is a toponym in apposition referring to the type locality.

##### Diagnosis.

This new species (Figs 1C–F, 2C–E) resembles *C.murinum* (Thorell, 1895) (Gravely 1931: 263, fig. 17A, B; Majumder and Tikader 1991: 72, figs 147, 148) and *C.duanbi* Yu & Li, 2020 (Zhang et al. 2020: 180, figs 3, 4A–D) in the general shape of palp and vulva, but can be distinguished from *C.murinum* by: 1) the shorter DTA; 2) copulatory ducts coiled around the spermathecae (vs. not encircling the spermathecae in *C.murinum*); and 3) lateral margin of the atrium close to the spermathecae (vs. away from the spermathecae in *C.murinum*), and from *C.duanbi* by: 1) the longer median apophysis and shorter cymbial spur; 2) DTA present (vs. absent in *C.duanbi*); 3) the wider copulatory ducts; and 4) atrial anterior margin absent (vs. arch-shaped in *C.duanbi*).

##### Description.

**Male (holotype)** (Figs 1A, B, 3A): Total length 9.38. Carapace 3.98 long, 2.88 wide; abdomen 5.40 long, 2.50 wide. Carapace pale yellow, with indistinct cervical grooves and radial grooves, cephalic region inconspicuously raised. All eyes with black rings, eye area colour slightly darker than carapace. AER slightly recurved, PER slightly wider than AER, slightly procurved in dorsal view. Eye sizes and interdistances: AME 0.17, ALE 0.18, PME 0.19, PLE 0.18; AME–AME 0.17, AME–ALE 0.27, PME–PME 0.27, PME–PLE 0.34. MOA 0.51 long, front width 0.53, back width 0.63. Chelicerae reddish brown, with four promarginal and three retromarginal teeth, with dense scopula in both margins. Clypeus height 0.08. Sternum orange, 1.84 long, 1.49 wide. Labium coloured as chelicerae, anterior edge clearly scopula, longer than wide. Endites yellowish brown. Legs yellowish, without distinct colour markings. Leg measurements: I 24.36 (4.80, 1.79, 8.08, 6.81, 2.88), II 14.95 (4.13, 1.13, 5.54, 2.97, 1.18), III 12.16 (3.63, 0.95, 3.47, 2.86, 1.25), IV 20.99 (5.72, 1.45, 5.15, 6.70, 1.97). Abdomen elongate-oval, dorsum with two pairs of muscular impressions and numerous grey patches, a pale narrow longitudinal band in middle, enclosed by grey freckles; venter with numerous dark grey spots. Spinnerets coniform, ALS larger and closer to each other; PMS smallest; PLS longest, with two segments, length of basal segment shorter than distal segment.

***Palp*** (Fig. 1C–F). Tibia long, c. ¾ of cymbium length. RTA short and sclerotized, shorter than ½ tibia length, with wide base and narrow apex, twisted around the axis from ventral view; DTA short and thin, stalk-shaped. Tip of cymbium long, c. ¾ of cymbium length. Cymbial fold well-developed and clearly visible in retrolateral view, c. ½ of cymbium length; cymbial spur beak-like, about same length as RTA. Tegulum oval, c. two times longer than wide, surface wrinkled. Median apophysis long, more than ½ of tegulum’s length, twisted around the axis. Embolus located on distal side of tegulum, at about 12 o’clock position, extending clockwise along tegular margin, curving to distal conductor. Conductor large, membranous, gradually tapering toward apex.

**Female (paratype)** (Figs 2A, B, 3B): Total length 9.68. Carapace 4.04 long, 2.96 wide; abdomen 5.64 long, 3.35 wide. Carapace reddish brown, with indistinct cervical grooves. Eye area colour slightly darker than carapace, both anterior and posterior eye rows recurved, PER slightly wider than AER. Eye sizes and interdistances: AME 0.17, ALE 0.16, PME 0.18, PLE 0.18; AME–AME 0.18, AME–ALE 0.38, PME–PME 0.34, PME–PLE 0.43. MOA 0.43 long, front width 0.55, back width 0.66. Chelicerae dark reddish brown, both margins with three teeth. Clypeus height 0.11. Sternum orange, 1.96 long, 1.57 wide. Labium coloured as chelicerae, almost equal in length and width. Leg measurements: I 19.29 (5.02, 1.45, 5.74, 4.76, 2.32), II 13.77 (3.91, 1.43, 3.79, 3.36, 1.28), III 10.56 (2.71, 1.31, 2.20, 3.08, 1.26), IV 14.41 (3.64, 1.44, 3.79, 4.04, 1.50). Abdomen oval, dorsum yellowish brown, with indistinct muscular impressions and narrow longitudinal band.

***Epigyne*** (Fig. 2C–E). Atrium large, located at middle portion of epigynal plate, filled with mating plug; arched atrial lateral margins are easily visible after removing the plug. Copulatory openings located at lateral margins of atrium. Copulatory duct visible through tegument of epigynal plate in ventral view. Spermathecae large, banana-shaped, c. two times longer than wide. Copulatory ducts coiled, forming about three ascending turns and then descending into the spermathecae. Fertilization ducts lamellar, broad, originate from posterior parts of spermathecae.

##### Distribution.

China (Yunnan).

#### ﻿ Cheiracanthium bifurcatum sp. nov.

Taxon classificationAnimaliaAraneaeCheiracanthiidae

0AC02D5B-FF8C-5638-B324-727DD51DD91A

https://zoobank.org/49DECA8D-DCB1-4E8B-8E3F-2EAC6BE63B30

**Figure 4.**
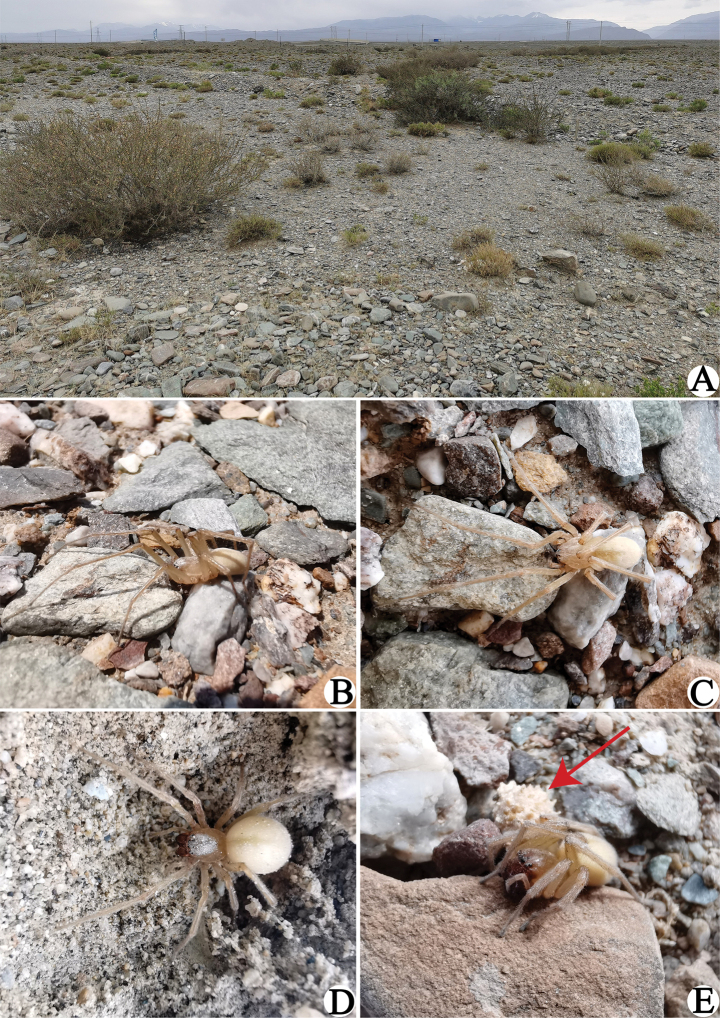
Habitat (**A**) and living specimens (**B–E**) of *Cheiracanthiumbifurcatum* sp. nov. **A** camelthorn steppe in Aksu **B, C** male holotype **D, E** female paratype, with arrow pointing to egg sac.

**Figure 5.**
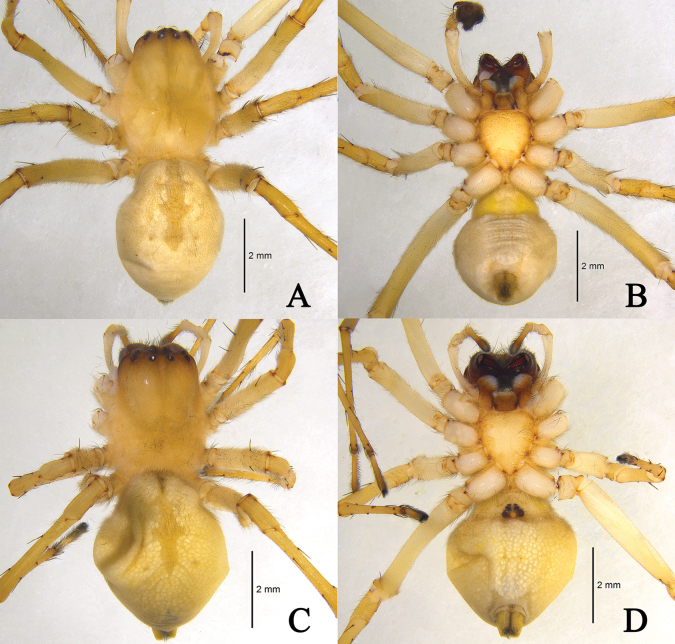
*Cheiracanthiumbifurcatum* sp. nov. **A** male holotype (ZYL604), dorsal view **B** same, ventral view **C** female paratype (ZYL605), dorsal view **D** same, ventral view.

**Figure 6.**
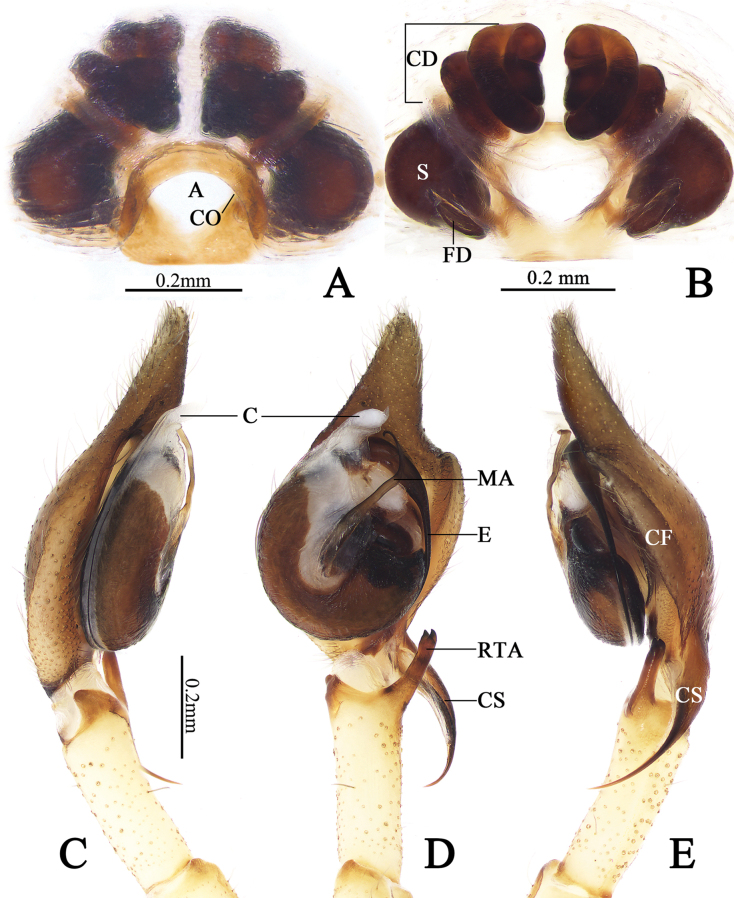
Copulatory organs of *Cheiracanthiumbifurcatum* sp. nov. **A** epigyne, ventral view **B** same, dorsal view **C** male left palp, prolateral view **D** same, ventral view **E** same, retrolateral view. Abbreviations: A = atrium, C = conductor, CD = copulatory duct, CF = cymbial fold, CO = copulatory opening, CS = cymbial spur, DTA = dorsal tibial apophysis, E = embolus, FD = fertilisation duct, MA = median apophysis, RTA = retrolateral tibial apophysis, S = spermatheca.

Figs 4 , 5 , 6 Chinese name: 双叉红螯蛛

##### Type material.

***Holotype*** ♂ (ZYL604), China: Xinjiang Uyger Autonomous Region, Aksu City, Wushi County, Yamansu Kirgiz Town, 41.070672°N, 78.840871°E, 1657 m elev., 26.V.2023, leg. Bo Liu. ***Paratype***: 1♀ (ZYL605), same data as holotype.

##### Etymology.

The specific epithet is an adjective from the Latin ‘bifurcate’, referring to the distally bifurcated retrolateral tibial apophysis in ventral view.

##### Diagnosis.

The male of this new species (Fig. 6C–E) is most similar to *C.japonicum* Bösenberg & Strand, 1906 (Vertyankin and Zaitsev 2022: 95, figs 9, 10), *C.brevispinum* Song, Feng & Shang, 1982 (Zhang et al. 2022: 180, fig. 132E–G) and *C.xinjiangense* Li & Zhang, 2023 (Li and Zhang 2023: 99, fig. 10C–E) by having biforked RTA, hook-shaped median apophysis and a triangular tip of cymbium, but can be distinguished from *C.brevispinum* by the longer cymbial spur (c. 0.8 times the length of tibia vs. 0.5 times in *C.brevispinum*), and from *C.japonicum* and *C.xinjiangense* by the nearly equal length of RTA’s two-pointed apex (vs. the prolateral apex longer than the retrolateral one in *C.japonicum* and shorter than the retrolateral one in *C.xinjiangense*). The female (Fig. 6A, B) is similar to *C.japonicum* Bösenberg & Strand, 1906 (Paik 1990: 5, figs 6–9), *C.exquestitum* Zhang & Zhu, 1993 (Li and Zhang 2024: 176, figs 4a, b, 5c, d) and *C.falcatum* Chen, Huang, Chen & Wang, 2006 (Chen et al. 2006: 12, fig. 2A, B) in having spiraling copulatory ducts and similarly shaped spermathecae, but can be distinguished by the 1:1 ratio of length to width of atrium (vs. 1:2 in other three species). Furthermore, it can be distinguished from *C.falcatum* by having four loops of copulatory ducts (vs. three loops in *C.falcatum*), from *C.exquestitum* by the fertilization ducts originating from the posterior parts of spermathecae (vs. median in *C.exquestitum*), and from *C.japonicum* by thinner transparent parts of copulatory ducts.

##### Description.

**Male (holotype)** (Figs 4B, C, 5A, B): Total length 7.58. Carapace 3.49 long, 2.82 wide; abdomen 4.09 long, 2.92 wide. Carapace pale yellow, with indistinct cervical grooves and radial grooves. All eyes with black rings, eye area colour slightly darker than carapace. AER slightly recurved, PER wider than AER, slightly procurved in dorsal view. Eye sizes and interdistances: AME 0.19, ALE 0.16, PME 0.15, PLE 0.17; AME–AME 0.15, AME–ALE 0.17, PME–PME 0.30, PME–PLE 0.32. MOA 0.56 long, front width 0.54, back width 0.59. Chelicerae reddish brown, with three promarginal and two retromarginal teeth, with dense scopula in both margins. Clypeus height 0.09. Sternum orange, 1.70 long, 1.55 wide. Labium and endites coloured as chelicerae, anterior edge clearly scopula. Legs yellowish. Leg measurements: I 19.91 (5.41, 1.49, 5.12, 5.74, 2.15), II 13.08 (3.10, 1.34, 3.25, 3.97, 1.42), III 10.36 (2.62, 1.21, 2.46, 3.00, 1.07), IV 16.02 (3.86, 1.45, 4.28, 4.77, 1.66). Abdomen oval, yellowish white, dorsum with indistinct muscular impressions and a dark longitudinal band; venter pale grey.

***Palp*** (Fig. 6C–E). Tibia long, c. ⅔ of cymbium length. RTA long, c. ½ of tibia’s length, finger-shaped, distally bifurcated. Cymbial furrow strongly developed and conspicuous, c. ⅔ of cymbium length; cymbial spur shorter than tibia length, tapering off into a filiform. Tegulum oval, c. 1.2× as long as wide. Median apophysis long, more than ½ of tegulum’s length, with a curved tip resembling a sickle in ventral view. Embolus located on distal side of tegulum, at about 11–12 o’clock position, extending clockwise along tegular margin, curving to distal conductor. Conductor large, membranous, gradually tapering toward apex.

**Female (paratype)** (Figs 4D, E, 5C, D): Total length 8.04. Carapace 3.66 long, 2.67 wide; abdomen 4.38 long, 3.39 wide. Carapace yellowish brown, with indistinct cervical grooves and radial grooves, cephalic region inconspicuously raised. Eye area colour slightly darker than carapace. AER slightly recurved, PER wider than AER, slightly procurved in dorsal view. Eye sizes and interdistances: AME 0.19, ALE 0.16, PME 0.13, PLE 0.15; AME–AME 0.24, AME–ALE 0.24, PME–PME 0.40, PME–PLE 0.42. MOA 0.51 long, front width 0.64, back width 0.65. Chelicerae dark reddish brown, with three promarginal and two retromarginal teeth. Clypeus height 0.09. Sternum orange, 1.74 long, 1.49 wide. Labium and endites reddish brown. Leg measurements: I 14.52 (3.81, 1.39, 3.43, 4.13, 1.76), II 9.88 (2.50, 1.16, 2.23, 3.00, 0.99), III 8.03 (1.99, 0.96, 1.81, 2.39, 0.88), IV 13.14 (3.29, 1.34, 3.28, 4.11, 1.12). Abdomen oval, dorsum with numerous yellow freckles and a dark longitudinal band; venter yellow, with numerous light spots in middle.

***Epigyne*** (Fig. 6A, B): Atrium sclerotized, located at posterior portion of epigynal plate, with arch-shaped anterior margin. Copulatory openings located at lateral margins of atrium. Copulatory duct and spermathecae visible through tegument of epigynal plate in ventral view. Spermathecae nearly pyriform, spaced by about 1.5 diameters, connected with spiral-coiled copulatory duct (each ascending portion of copulatory duct coils forming three entwined loops and then form one descending coil and downward leading to spermatheca). Fertilization ducts lamellar, originate from posterior parts of spermathecae, extending anterolaterally.

##### Distribution.

China (Xinjiang).

##### Habitat.

All specimens were found under stones in a very flat area with numerous crushed stones and covered with prickly bushes, such as camelthorn and tamarisks, reaching about 30–50 cm in height (Fig. 4).

## Supplementary Material

XML Treatment for Cheiracanthium

XML Treatment for Cheiracanthium bannaensis

XML Treatment for Cheiracanthium bifurcatum

## ﻿Additional information

### Author ORCIDs

Zhaoyi Li https://orcid.org/0000-0001-6303-6041

Feng Zhang https://orcid.org/0000-0002-3347-1031

### Data availability

All sequences were deposited in GenBank, and other data that support the findings of this study are available in the main text.
